# Clinical characteristics of COVID-19 patients with clinically diagnosed bacterial co-infection: A multi-center study

**DOI:** 10.1371/journal.pone.0249668

**Published:** 2021-04-05

**Authors:** Shengyang He, Wenlong Liu, Mingyan Jiang, Peng Huang, Zhi Xiang, Dingding Deng, Ping Chen, Lihua Xie

**Affiliations:** 1 Department of Respiratory and Critical Care Medicine, The Third Xiangya Hospital of Central South University, Hunan, China; 2 Department of Respiratory and Critical Care Medicine, The Second People Hospital of Yueyang, Yueyang, China; 3 Department of Respiratory and Critical Medicine, Xiangtan Central Hospital, Xiangtan, China; 4 Department of Respiratory and Critical Medicine, The Zhuzhou Central Hospital, Zhuzhou, China; 5 Department of Respiratory and Critical Medicine, The First People Hospital of Huaihua, Huaihua, China; 6 Department of Respiratory and Critical Medicine, The First People Hospital of Shaoyang, Shaoyang, China; 7 Department of Respiratory and Critical Care Medicine, The Second Xiangya Hospital of Central South University, Hunan, China; Montana State University, UNITED STATES

## Abstract

**Objective:**

To understand the clinical characteristics of COVID-19 patients with clinically diagnosed bacterial co-infection (CDBC), and therefore contributing to their early identification and prognosis estimation.

**Method:**

905 COVID-19 patients from 7 different centers were enrolled. The demography data, clinical manifestations, laboratory results, and treatments were collected accordingly for further analyses.

**Results:**

Around 9.5% of the enrolled COVID-19 patients were diagnosed with CDBC. Older patients or patients with cardiovascular comorbidities have increased CDBC probability. Increased body temperature, longer fever duration, anhelation, gastrointestinal symptoms, illness severity, intensive care unit attending, ventilation treatment, glucocorticoid therapy, longer hospitalization time are correlated to CDBC. Among laboratory results, increased white blood cell counting (mainly neutrophil), lymphocytopenia, increased procalcitonin, erythrocyte sedimentation rate, C-reaction protein, D-dimer, blood urea nitrogen, lactate dehydrogenase, brain natriuretic peptide, myoglobin, blood sugar and decreased albumin are also observed, indicating multiple system functional damage. Radiology results suggested ground glass opacity mixed with high density effusion opacities and even pleural effusion.

**Conclusion:**

The aged COVID-19 patients with increased inflammatory indicators, worse lymphopenia and cardiovascular comorbidities are more likely to have clinically diagnosed bacterial co-infection. Moreover, they tend to have severer clinical manifestations and increased probability of multiple system functional damage.

## Introduction

The Corona Virus Disease 2019 (COVID-19), caused by the severe acute respiratory syndrome coronavirus-2 (SARS-CoV-2), has affected most countries all over the world since its first case in end of 2019. The genomic characteristics of SARS-CoV-2 was initially reported by Lu and colleagues, suggesting this coronavirus had enveloped RNA, resembling severe acute respiratory syndrome coronavirus (SARS-CoV) in both structural and homological ways [1]. To date, there are more than 10 million confirmed cases, with over 500 thousand reported deaths worldwide, according to the World Health Organization (WHO) COVID-19 daily report dashboard (https://covid19.who.int/). As reported by the WHO, North America has been the most affected continent, followed by Europe. China has undergone this viral crisis and successfully blocked it’s spread to some extent. However, there still have already been more than 80 thousand confirmed cases and 4,600 deaths in China.

During this COVID-19 attack, besides the primary infection of SARS-CoV-2, many other complications are emerging, contributing greatly to the mortality. Among these, co-infection plays a crucial role, threatening many COVID-19 patients’ lives [2]. As were reported by researchers, the prevalence of co-infection was variable, being found occurred in half of the non-survivors [3]. The pathogens of respiratory co-infection could be many, either common or rare, including bacteria, virus, fungus, etc. And bacteria was reckoned one of the most commonly isolated one [4]. There is no doubt that the co-infection could be a significant promoter to the final mortality of COVID-19 patients. However, during the outbreak of COVID-19, many hospital and health care organizations are short of hands, and many potential diagnoses could not be made according to the “golden diagnostic criteria”. The concept of “clinically diagnosed bacterial co-infection” was therefore presented in this study in order to give more indications toward the timing of launching empiric anti-bacterial therapy, when patients are highly considered to have suspected bacterial co-infection.

To decrease the mortality of COVID-19 patient as much as possible, the early recognition and managements of bacterial co-infection seem rather indispensable. In the present study, 905 confirmed COVID-19 patients from 7 different cities in both Hunan and Hubei province, China, were enrolled for investigation. The cases with suspected bacterial co-infection were clinically diagnosed according to some specific diagnostic criteria (see below). The clinical data of these clinically diagnosed bacterial co-infection (CDBC) patients were collected for further retrospective analyses in order to throw light upon the characteristics of them, facilitating early recognition and managements.

## Method

### Study design and participants

The initial hospital admittance time of the enrolled patients range from January 10^th^ to February 28^th^, 2020. Every participant of this study has either signed written or made oral consensus (the aims of the conducting study, the privacy protection policy, the duties and rights of enrolled patients were all told before oral consensus was made and the oral consensus was recorded by a voice recorder), as we were short of health personnel to make every participant sign a written consent during the viral outbreak. And this was permitted by the Ethics Committee of The Second Xiangya Hospital of Central South University. COVID-19 diagnoses were made according to the diagnostic criterion from the *7th version of the guidelines on the Diagnosis and Treatment of COVID-19* by the National Health Commission of China. All the clinical data were retrospectively recorded from the electronic medical record system in 7 different cities’ central general hospital respectively, including Yueyang, Shiyan, Shaoyang, Zhuzhou, Huaihua, Huanggang and Loudi. This study was approved by the Ethics Committee of The Second Xiangya Hospital of Central South University and was performed in accordance with the principles of the Helsinki Declaration II.

### Data collection

All the 905 enrolled patients were from the separated COVID-19 units of these 7 hospitals. Computer tomography (CT) scan evaluations were made by at least 2 specialists from the radiology department. The SARS-CoV-2 nucleic acid RT-PCR test quality control was performed by specialists from the clinical laboratory department. The collected clinical data include: the demographic descriptions, main symptoms, comorbidities, regular laboratory results (e.g. blood routine examination, renal function, liver function, myocardial enzyme), main treatments, etc. To be specific, all the laboratory and radiological results, from the beginning to the end, of those CDBC patients were collected when the bacterial co-infection diagnoses were clinically made (diagnostic criteria were described below). However, the test results of those non-CDBC patients were collected with the most abnormal ones during the whole hospitalization period. For privacy reasons, the raw data of these patients are not presented.

### Clinical definitions

COVID-19 diagnosis was made by SARS-CoV-2 RNA RT-qPCR test with nasopharyngeal swabs before hospitalization of each participant. The RNA detection kits were provided by Sansure Biotech (Changsha, China) and being manipulated by specialized clinical laboratory technicians according to the manufacturer’s protocol.

The clinical diagnosis criteria of bacterial co-infection are as follow (patients meet all of these criteria shall be considered CDBC): a, newly increased WBC counting (>9.5*10^9/L), with a majority of neutrophil; b, newly increased airway purulent secretion; c, increased serum procalcitonin; d, typical peripheral ground glass opacity mixed with increased density effusion opacities; e, effective empiric anti-bacterial therapy.

COVID-19 patients who could be considered discharged when meeting all the discharging criteria from the 7^th^ version of the *Guidelines on the Diagnosis and Treatment of COVID-19* by the National Health Commission of China. Briefly, (1) normal body temperature for more than 3 days; (2) significantly recovered respiratory symptoms; (3) lung imaging shows obvious absorption and recovery of acute exudative lesion; (4) negative results of the nucleic acid tests of respiratory pathogens for consecutive two times (sampling interval at least 1 day).

Mechanical ventilation indications: a, failed high-flow nasal cannula (HFNC) or non-invasive positive pressure ventilation (NIPPV) therapy when 300>PaO2/FiO2≥150; b, PaO2/FiO2≤150 with failed short term NIPPV.

### Statistics analysis

The continuous variables were denoted as median (interquartile range, IQR) and comparisons were performed using the Mann-Whitney test. The categorical variables were denoted as n (%) and compared by using the Chi-square test or Fisher’s exact Chi-square test. The association between potential risk factors and outcomes was estimated by using logistic regression. Kaplan–Meier methods were used for survival curve plotting and examined by log-rank test. The association between clinically diagnosed bacterial co-infection and all-cause mortality was examined by using multivariate Cox regression model. All analyses were performed using R software (The R Foundation, http://www.r-project.org, version 3.6.1). A two-sided significance level of 0.05 was used to evaluate statistical significance.

## Results

### Demographic features and clinical symptoms

All the enrolled 905 confirmed COVID-19 patients were from 7 different centers (Yueyang, Shiyan, Shaoyang, Zhuzhou, Huaihua, Huanggang and Loudi). 86 out of 905 patients (9.5%) were CDBC according to our diagnostic criteria described above. Among the basic demographic features, CDBC patients tend to be older and the rest were found no differences (Table 1). Fever was more common among CDBC patients, especially the cases over 39°C, moreover, anhelation, fatigue, gastrointestinal symptoms and respiratory rate were all increased among CDBC patients, while SPO2 was decreased (Table 1).

**Table 1:** Demographic features, clinical symptoms and physical signs.

	All patients	COVID-19		P value
		Without CDBC	With CDBC	
**Total**	905	819	86	
**Age**	47 (35–57)	46 (34–56)	62 (49–75)	<0.001
**Gender**				0.007
** Female**	463 (51%)	431 (53%)	32 (37%)	NA
** Male**	442 (49%)	388 (47%)	54 (63%)	NA
**BMI**	23 (21–25)	23 (21–25)	23 (21–26)	0.88
**Fever**	672 (74%)	595 (73%)	77 (90%)	<0.001
**Cough**	714 (79%)	649 (79%)	65 (76%)	0.43
**Sputum**	424 (47%)	376 (46%)	48 (56%)	0.08
**Hemoptysis**	16 (2%)	12 (1%)	4 (5%)	0.06
**Anhelation**	134 (15%)	92 (11%)	42 (49%)	<0.001
**Fatigue**	425 (47%)	378 (46%)	47 (55%)	0.13
**Muscle soreness**	142 (16%)	129 (16%)	13 (15%)	0.88
**Headache**	48 (5%)	44 (5%)	4 (5%)	>0.99
**Gastrointestinal symptoms**	117 (13%)	93 (11%)	24 (28%)	<0.001
**Heart rate**	81 (75–90)	81 (75–90)	86 (76–95)	0.06
**Respiratory rate**	20 (19–20)	20 (19–20)	20 (20–23)	<0.001
**SPO2**	98 (96–99)	98 (97–99)	95 (93–96)	<0.001
**SBP**	125 (120–132)	124 (119–130)	128 (120–140)	0.02
**DBP**	80 (72–84)	80 (72–84)	80 (73–87)	0.33
**Highest temperature**	37.8 (37.0–38.5)	37.7 (37.0–38.5)	38.5 (37.6–39.0)	<0.001
** <38°C**	473 (52%)	447 (55%)	26 (30%)	<0.001
** 38–39°C**	375 (41%)	335 (41%)	40 (47%)	
** >39°C**	57 (6%)	37 (5%)	20 (23%)	
**Duration of fever**	8 (5–12)	8 (5–12)	10 (6–14)	0.03

### CDBC occurred more frequent among COVID-19 patients with cardiovascular comorbidities

We analyzed several common chronic disease types among the enrolled patients. Hypertension and coronary heart disease are more prevalent among CDBC patients, along with diabetes. However, chronic lung diseases, liver diseases, kidney diseases and tumor were not different between CDBC and no-CDBC patients (Table 2).

**Table 2:** Comorbidities of COVID-19 patients.

	All patients	COVID-19		P value
		Without CDBC	With CDBC	
**Hypertension**	141 (16%)	105 (13%)	36 (42%)	<0.001
**Diabetes**	84 (9%)	71 (9%)	13 (15%)	0.05
**Coronary heart disease**	37 (4%)	29 (4%)	8 (9%)	0.01
**Chronic lung disease**	27 (3%)	22 (3%)	5 (6%)	0.17
**Liver disease**	8 (1%)	8 (1%)	0 (0%)	>0.99
**Kidney disease**	8 (1%)	6 (1%)	2 (2%)	0.17
**Tumor**	11 (1%)	9 (1%)	2 (2%)	0.28

### COVID-19 patients with CDBC tend to have increased neutrophils count and decreased lymphocytes

Generally speaking, COVID-19 patients have normal WBC counting (20% of all the enrolled COVID-19 patients had increased WBC count). However, according to our findings, once WBC of COVID-19 patients increased notably, with a majority of neutrophils, they were highly suspected to have bacterial co-infection (43% of the CDBC had increased WBC count while non-CDBC only 3%). Moreover, lymphocytes count could even be further decreased (Table 3).

**Table 3:** Blood routine of COVID-19 patients.

	All patients	COVID-19		P value
		Without CDBC	With CDBC	
WBC (*10^9)	5.0 (3.9–6.7)	4.9 (3.8–6.2)	10.5 (6.0–12.6)	<0.001
>10*10^9/L, n(%)	179 (20%)	21 (3%)	37 (43%)	<0.001
(4–10)*10^9/L, n(%)	668 (74%)	628 (77%)	40 (47%)	0.007
<4*10^**9**^**/L, n(%)**	58 (6%)	170 (21%)	9 (10%)	
**Neutrophil**	3.6 (2.4–8.6)	3.3 (2.3–6.6)	10.7 (7.9–14.6)	<0.001
**Lymphocyte**	1.2 (0.8–1.6)	1.2 (0.8–1.6)	0.6 (0.4–1.0)	<0.001
<1*10^9/L, n(%)	166 (18%)	135 (16%)	31 (36%)	<0.001
(1–1.5)*10^9/L, n(%)	595 (66%)	545 (67%)	50 (58%)	0.43
>1.5*10^9/L, n(%)	144 (16%)	139 (17%)	5 (6%)	0.08
**Hemoglobin**	190 (160–240)	130 (119–144)	131 (118–147)	0.96
**Platelet**	190 (160–240)	201 (154–257)	180 (131–258)	0.2

### Serum inflammatory indicators and coagulation, hepatic and renal function

Procalcitonin (PCT) of CDBC patients were found notably increased, supporting its widely accepted diagnostic role for bacterial infection. Erythrocyte sedimentation rate (ESR) and C-reaction protein (CRP), common inflammatory indicators as they are, were both increased in CDBC patients. In blood clotting indexes, prothrombin time (PT) and D-dimer were found increased compared with non-CDBC patients, along with aspartate amino transferase (AST) and bilirubin in liver function tests. Moreover, blood urea nitrogen (BUN), lactate dehydrogenase (LDH), brain natriuretic peptide (BNP), Myohemoglobin and random blood sugar (RBS) were all increased in CDBC patients (Table 4).

**Table 4:** Inflammatory indictors and other laboratory results.

	All patients	COVID-19		P value
		Without CDBC	With CDBC	
**Procalcitonin**	0.05 (0.05–0.10)	0.05 (0.04–0.10)	0.29 (0.10–0.57)	<0.001
** <0.5, n(%)**	886 (98%)	811 (99%)	75 (87%)	<0.001
** > = 0.5, n(%)**	19 (2%)	8 (1%)	11 (13%)	0.007
**ESR**	29.0 (13.0–48.0)	28.0 (12.0–45.8)	46.3 (28.0–82.0)	0.002
**CRP**	7.2 (5.0–23.8)	6.0 (5.0–16.2)	59.2 (21.0–110.1)	<0.001
**PT**	12.0 (10.8–12.9)	11.8 (10.8–12.8)	13.4 (12.4–14.6)	<0.001
**APTT**	31.4 (27.3–35.7)	31.6 (27.4–35.8)	29.4 (25.6–34.8)	0.22
**D-dimer**	0.3 (0.2–0.6)	0.3 (0.2–0.5)	1.8 (0.6–4.1)	<0.001
**ALT**	23 (16–41)	22 (15–40)	28 (21–55)	0.007
**AST**	24 (19–34)	22 (18–32)	36 (25–56)	<0.001
**CK**	60.0 (41.5–96.5)	58.0 (40.5–93.5)	73.5 (46.0–159.4)	0.09
**CK-MB**	11.0 (4.0–38.5)	10.5 (2.6–35.4)	14.0 (8.0–51.5)	0.09
**bilirubin**	12.7 (8.7–20.8)	12.3 (8.7–19.5)	24.3 (10.6–33.8)	<0.001
**K**^**+**^	4.3 (3.9–4.6)	4.3 (3.9–4.6)	4.3 (3.8–5.2)	0.38
**Na**^**+**^	139.3 (137.5–141.2)	139.2 (137.7–141.0)	139.8 (136.4–143.8)	0.18
**BUN**	12.7 (8.7–20.8)	3.9 (3.2–4.8)	7.8 (4.4–16.4)	<0.001
**Creatinine**	67.3 (55.1–81.4)	66.0 (55.0–80.2)	74.8 (62.6–113.2)	0.002
**LDH**	190 (160–240)	183 (160–226)	337 (241–510)	<0.001
**TNI**	0.01 (0.01–0.03)	0.01 (0.01–0.03)	0.03 (0.02–0.03)	0.001
**BNP**	148.0 (25.0–420.5)	99.0 (18.0–251.9)	427.0 (156.7–706.0)	<0.001
**Albumin**	39.2 (33.3–43.6)	40.3 (34.9–44.0)	30.3 (28.5–33.6)	<0.001---
**Myohemoglobin**	35.0 (30.0–62.8)	31.0 (30.0–51.0)	119.9 (67.7–379.6)	<0.001
**RBS**	5.4 (4.9–6.7)	5.3 (4.9–6.0)	8.3 (6.5–10.6)	<0.001

### Radiological findings and treatments

**Fig 1:**
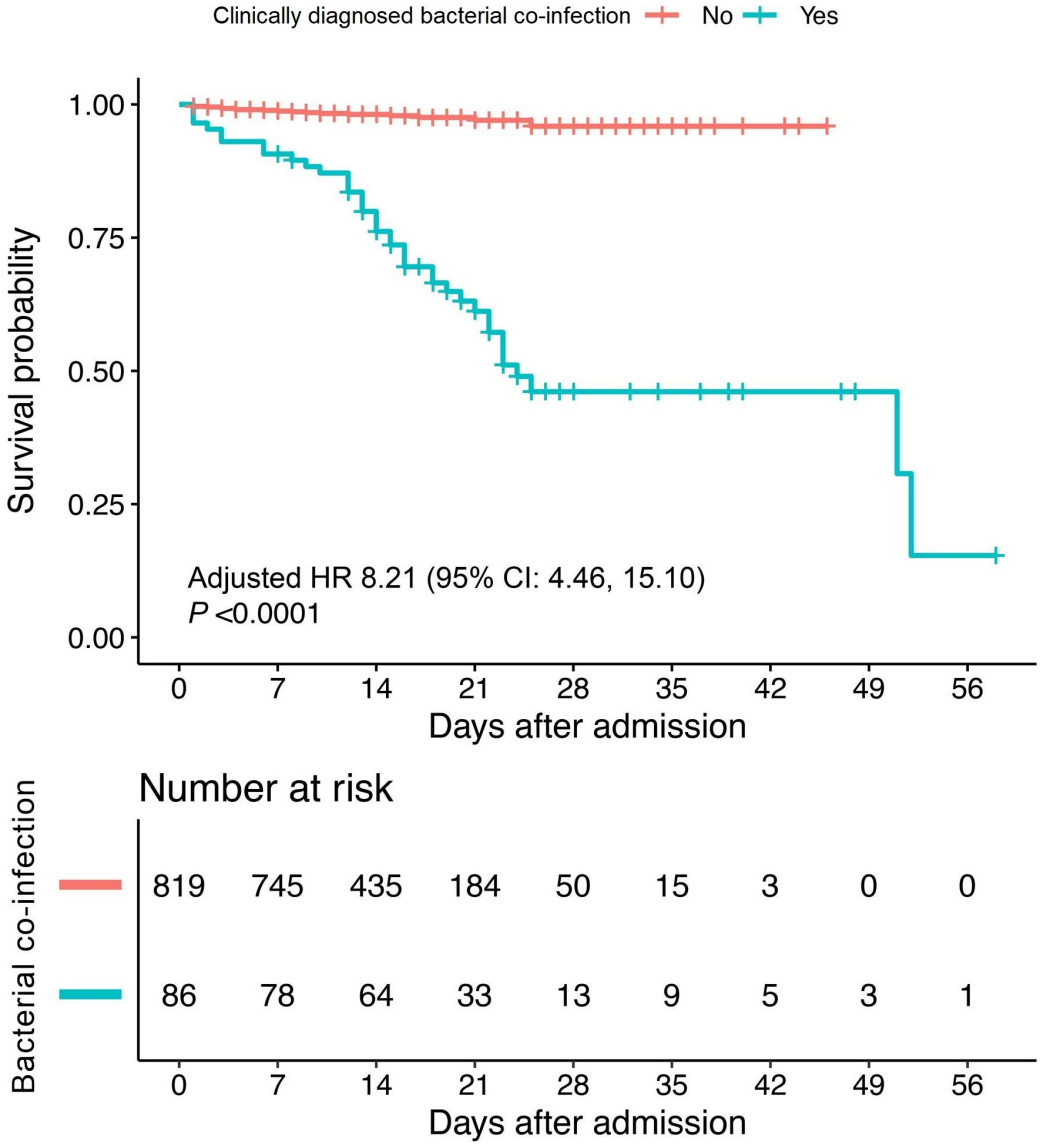
Kaplan-Meier curves for cumulative survival probability during hospitalization. The red curve indicated non-CDBC patients and the blue-green curve indicated CDBC patients. The survival curves were plotted based on the day of hospital admittance (day 0). We further performed Cox regression analysis to investigate the association between CDBC and risk of in-hospital mortality with adjustment of confounding factors, including gender, age and pre-existing comorbidities. Compared with non-CDBC patients, those with CDBC were subjected to a higher risk of mortality with a hazard ratio (HR) 8.21 (95% CI: 4.46 to 15.10), that is, patients with CDBC have 721% more death risk than the non-CDBC.

Every enrolled patient had at least 2 CT scan results during hospitalization. CDBC patients always have ground-glass opacities (GGO) mixed with high density effusion opacities (HDEO), and pleural effusion could even be found in some CDBC cases (Table 5). As for treatments, CDBC patients tend to be in need of more critical care facilities, including ventilation and other ICU medical care therapies. Also, the application of glucocorticoid was more common among CDBC patients. Moreover, retrospectively, we found the total mortality was notably increased among those CDBC patients (Table 5, Fig 1).

**Table 5:** Radiological findings and treatments.

	All patients	COVID-19		P value
		Without CDBC	With CDBC	
**Radiology**				
** GGO**	523 (58%)	495 (60%)	28 (33%)	<0.001
** HDEO**	6 (1%)	4 (0%)	2 (2%)	0.1
** Mixed**	30 (3%)	24 (3%)	6 (7%)	0.046
** PE**	36 (4%)	27 (3%)	9 (10%)	0.001
**Treatments**				
** ICU, n (%)**	53 (6%)	19 (2%)	34 (40%)	<0.001
** Mechanical ventilation**	66 (7%)	19 (2%)	47 (55%)	<0.001
** Hospitalization duration**	15 (10–20)	14 (10–20)	18 (13–24)	<0.001
** In-hospital mortality, n (%)**	57 (6%)	18 (2%)	39 (45%)	<0.001
** Application of glucocorticoid, n (%)**	224 (25%)	161 (20%)	63 (73%)	<0.001

Ground-glass opacity (GGO); High density effusion opacities (HDEO); Pleural effusion (PE).

### Other potential predictors for CDBC diagnosis

To evaluate the risk factors of CDBC, a logistic regression analysis was conducted. Decreased albumin account and glucocorticoid treatment could be the best predictors for CDBC, with the sensitivities 82% and 73% respectively. Moreover, advanced age, high WBC account, lymphopenia, PCT and CRP could all be CDBC predictors with reasonably high sensitivities. The cardiovascular comorbidities also could be considered predictors, however, the sensitivity is relatively low (Table 6).

**Table 6:** Various predictors of the occurrence of clinically diagnosed bacterial co-infection in COVID-19 patients.

Variables	OR (95% CI)	AUC(95% CI)	P-value	Sensitivity
**Age**	1.06 (1.04, 1.07)	0.73 (0.67, 0.79)	<0.001	0.58
**WBC**	1.58 (1.43, 1.75)	0.81 (0.74, 0.87)	<0.001	0.63
**Lymphocyte**	0.10 (0.04, 0.24)	0.77 (0.69, 0.86)	<0.001	0.66
**PCT**	5.11 (1.98, 13.19)	0.83 (0.76, 0.91)	<0.001	0.66
**CRP**	1.03 (1.02, 1.05)	0.83 (0.75, 0.91)	<0.001	0.68
**Comorbidity**				
**Chronic lung disease**	2.24 (0.82, 6.06)	0.51 (0.49, 0.54)	0.11	0.06
**Diabetes**	1.88 (0.99, 3.55)	0.53 (0.49, 0.57)	0.053	0.15
**Tumor**	2.14 (0.46, 10.08)	0.51 (0.49, 0.52)	0.33	0.02
**Cardiovascular disease**	2.79 (1.23, 6.32)	0.53 (0.50, 0.56)	0.014	0.09
**Albumin**	0.79 (0.74, 0.85)	0.84 (0.78, 0.90)	<0.001	0.82
**Glucocorticoid**	11.19 (6.74, 18.60)	0.77 (0.72, 0.82)	<0.001	0.73
**Attandence of ICU**	27.53 (14.70, 51.57)	0.69 (0.63, 0.74)	<0.001	0.4
**Mechanical ventilation**	50.74 (27.23, 94.54)	0.76 (0.71, 0.81)	<0.001	0.55

## Discussion

Up to date, few studies have reported the clinical characteristics of COVID-19 patients with bacterial co-infection, however, co-infection of influenza virus [5], fungus [6] and other atypical respiratory pathogens (e.g. mycoplasma) [7] have all been reported. According to our study, around 9.5% COVID-19 people were CDBC, which could give a huge rise to the whole mortality. A multi-center study was therefore conducted to analyze the clinical characteristics of the CDBC cases among COVID-19 patients, finding them have more severe illness and increased inflammatory activities, along with an increased multi-system functional damage tendency.

Viruses are one of the most common respiratory pathogens, and bacterial co-infection was not rare during primary respiratory viral infection, including influenza of many types (e.g. H1N1, H7N9, etc) [8–10], RSV [11], rhinovirus [12], and parainfluenza [13], etc) Specifically, a study regarding H1N1 in America showed 207 out of 683 enrolled patients acquired respiratory bacterial infection symptoms 72h after hospitalization, with 5.2 days as an average onset time. More than 60% of H1N1 patients had suspected bacterial co-infection, and around 30% patients could be diagnosed with bacterial co-infection [14]. Moreover, another study concerning critical influenza cases indicated that once bacterial co-infection occurred, the hospitalization duration and the final mortality would be both extremely increased [15]. Furthermore, bacterial coinfection could also be found during infection with other corona virus family members. During the 2003 SARS crisis, CDBC was a major promoter of high mortality, especially among those critical cases [16]. Moreover, bacterial co-infection in MERS was also notable, increasing the illness severity, and low albumin could be one of the most important risk factors [17, 18], which is similar with our findings. However, the coinfected bacterial pneumonia cases were up to 31%, not to mention the other types of bacterial co-infection. In this COVID-19 pandemic, Yang and colleagues have reported that hospital-acquired pneumonia (HAP) was found in around 11.5% COVID-19 patients, which is in line with our results (9.5%), and bacterial co-infection is a major inducer of death as it could eventually lead to many organs and system failures, including sever bacterial pneumonia, sepsis, and bacterial meningitis [19]. Also, many other studies reconfirmed the vital phenomenon that the antibiotic resistance could occur in COVID-19 patients with bacterial co-infection [20–22].

According to our results, COVID-19 patients typically present with a normal white blood cell count, however, a suddenly increased WBC count during hospitalization shall alert the occurrence of bacterial co-infection. Lymphopenia, which could be resulted from a severe immune system disorder, is common in COVID-19 patients [5]. The cause of lymphopenia in COVID-19 patients is widely considered cytokine release syndrome (CRS) by researchers [23]. According to the studies regarding SARS-CoV and MERS-CoV, dendritic cells are always infected by the former, while T cells and monocytes the latter via dipeptidyl peptidase 4 (DPP4) [24, 25]. Similarly, it is possible that SARS-CoV-2 would also affect dendritic cells, and therefore leading to the release of many cytokines and T cell apoptosis, eventually leading to immune system disfunction [24, 26]. This could be a key to the occurrence of CDBC as we found the lymphocyte count was further decreased in CDBC patients, indicating immune system disorders with a higher level. The increased inflammatory activity is a feature of COVID-19 [23], and the CDBC patients were found with further increased inflammation levels as CRP and ESR were notably ascendant [2], which was in line with the present study. PCT could be one of the most effective diagnostic indicators, which is also one of our CDBC diagnosis criteria, and studies showed a normal PCT level makes bacterial infection less likely and could guide discontinuation of anti-bacterial co-infection therapy, however, an increased PCT level is of great sensitivity but less specificity for bacterial infection diagnosis as it could also sometimes be increased in some other clinical symdromes (e.g. acute renal failure) [27]. However, we noticed an increased CRP and ESR rate among CDBC patients, and a study reported that the combination of increased PCT, CRP could provide a more useful method of distinguishing CDBC from an H1N1 influenza infection alone [28]. Segmental or lobar focal dense consolidation ± ground-glass opacities was reported the radiological feature of COVID-19 patients with bacterial co-infection [2], and these results are also similar to our findings in CDBC patients’ CT scan. Increased PT, AST, CK-MB, bilirubin, BUN, creatinine, TnI, LDH and myohemoglobin all together indicated a multi-system function damage and this might result from a secondary bacterimia following CDBC. Still, some researchers argued that the use of corticosteroid may have potential risks, as it could suppress our immune functions [29], which might therefore contribute to CDBC. We also found COVID-19 patients with CDBC tend to have an increased corticosteroid utility rate. Intriguingly, in the present study, we found older COVID-19 patients were more likely to have CDBC, while some other studies found CDBC was not age associated [30]. A potential reason could be that SARS-CoV-2 would induce more drastic immune reaction (e.g. CRS) than other respiratory viral infection [23], and immune function could be less active among older patients which might eventually give rise to the CDBC possibility. With the survival curve, we retrospectively noticed that, CDBC patients have increased possibility of death as the co-infection would aggravate the inflammatory burden of the body and toxic side effects of major organs (Kidney and liver, manly caused by antibiotics therapy).

Some limitation of the present study merit consideration. Initially, it is a real-world retrospective study, and the data collected were limited. A prospective analysis could be of more confidence and less bias. Secondly, the timing of CDBC onset was not distinguished, that is, CDBC could be community, hospital acquired or ventilation associated. And this might make the results less specific to HAP. Moreover, due to the lack of understanding of this highly contagious novel virus, the pathogens of CDBC have not been identified universally for safety reasons at the beginning of the global pandemic. But currently, microbial cultivation of respiratory secretions were recommended to make studies more informative and guiding the antibiotic therapy accordingly. Furthermore, a collection of dynamic laboratory and radiological findings could provide more confidence in the results.

To summarize, 86 out of 905 (9.5%) confirmed COVID-19 patients were CDBC according to our diagnosis criteria (see methods part). CDBC patients tend to be more aged, and having more severe clinical manifestations with increased WBC account and worse lymphopenia. Inflammatory indicators are increased, along with increased biochemical laboratory results indicating multi-system function damage tendency. And GGO mixed with HDEO could be the feature of CDBC radiological results. Moreover, advanced age, high WBC account, lymphopenia, PCT, CRP, cardiovascular comorbidities, the utilities of glucocorticoid and ventilation are all risk factors of CDBC. To better identify and manage CDBC in early stage, more prospective studies regarding CDBC are extremely necessary.
